# A case of surgery for congenital esophagobronchial fistula accompanied by a destroyed lung

**DOI:** 10.1186/s40792-016-0221-y

**Published:** 2016-09-08

**Authors:** Masaki Ikeda, Yoshitake Murata, Ryoko Ohnishi, Tatsuo Kato, Akira Hara, Takuji Fujinaga

**Affiliations:** 1 Department of Thoracic Surgery, Nagara Medical Center, Nagara 1300-7, Gifu, 502-8558 Japan; 2 Department of Respiratory Medicine, Nagara Medical Center, Gifu, Japan; 3 Department of Tumor Pathology, Gifu University Graduate School of Medicine, Gifu, Japan

**Keywords:** Congenital esophagobronchial fistula, Pulmonary sequestration, Destroyed lung, Repeated pneumonia, Bronchiectasis

## Abstract

Congenital esophagobronchial fistula (EBF) is rarely seen in adults. We report a case of EBF detected in adulthood with a destroyed lung. A 67-year-old man experienced repeated pneumonia during his childhood. Since the age of 38, he had often suffered from bloody phlegm and always had a cough and sputum during oral intake. Before cardiac surgery for atrial fibrillation and valvular disease, computed tomography (CT) detected bronchiectasis, which could cause pulmonary bleeding during heart surgery, and the patient was introduced to our hospital for lung resection. A fistula between the esophagus and the right lower lung lobe was found using CT, esophagoscopy, and esophagography. Contrast CT and angiography revealed an abnormal artery branching from the inferior phrenic artery into the lobe. As indicated by intraoperative findings, the middle and lower lobes had strongly adhered to chest wall and diaphragm, but we located the fistula easily without adhesion to the surroundings, severed it using an automatic stapler, and resected the middle and lower lobes. The symptoms disappeared immediately, and the patient was uneventfully discharged.

The diagnosis of congenital EBF was established with intraoperative findings and pathological exam. The existence of pulmonary sequestration was suggested because of the long-term absence of any symptoms during his adulthood, the tract of the EBF running into the lung, not directly into the bronchus, and a septum pathologically detected in the right lower lobe. A congenital EBF should be considered for differential diagnosis in cases of limited bronchiectasis in elderly people.

## Background

Congenital esophagobronchial fistula (EBF) is mostly found in children because it is almost always accompanied by esophageal atresia [1]. We report a case of a congenital EBF in an adult with a destroyed lung.

## Case presentation

**Fig. 1:**
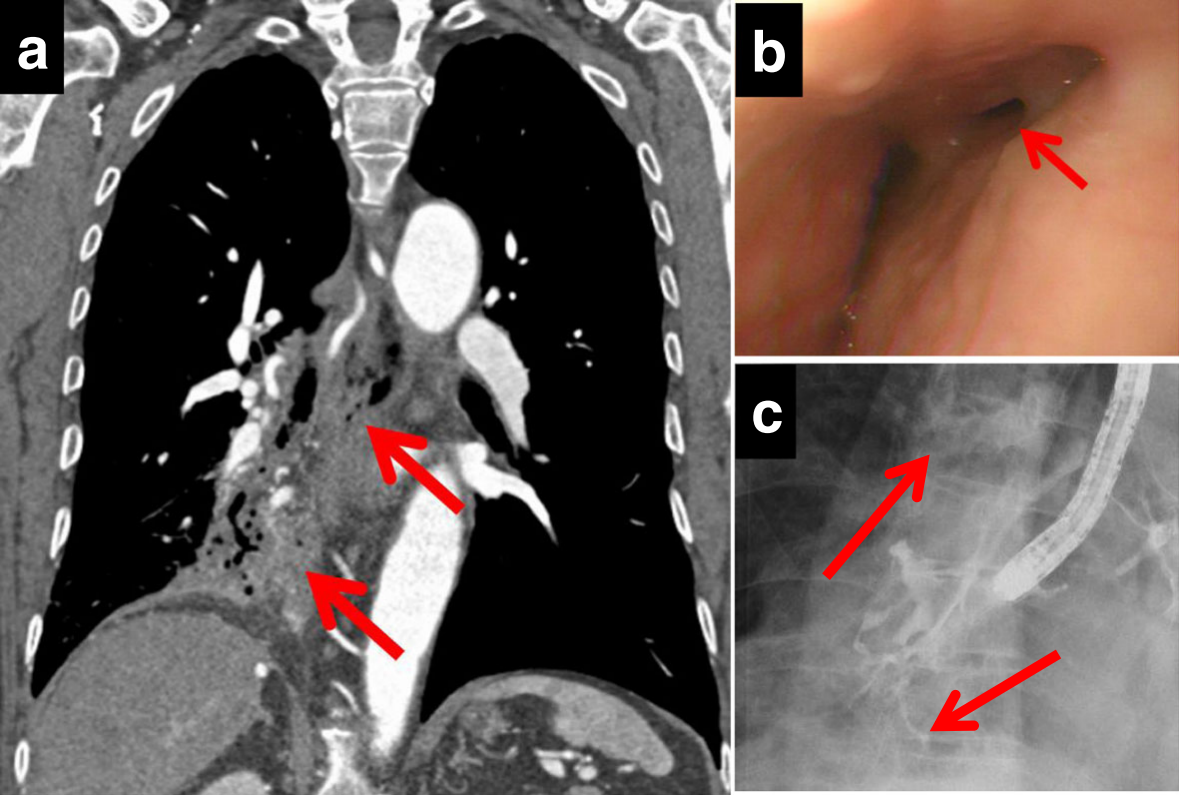
**a** Chest contrast-computed tomography. A connection (*arrows*) between the middle of the esophagus and the right lower lung lobe was suspected. **b** Esophagoscopy findings. A diverticulum-like depression was seen (*arrow*) at the 2 o’clock position on the wall of the middle esophagus. **c** Esophagography findings. The flow of the contrast medium was detected (*arrow*) from the esophagus depression to the right lower lung and finally to the right main bronchus

**Fig. 2:**
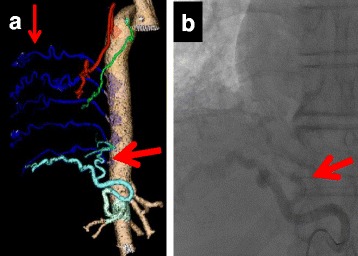
**a** CT angiography. An abnormal artery derived from the lower phrenic artery (*thick arrow*). Several developed bronchial arteries (*thin arrow*) from the descending aortic artery were detected. **b** Catheter angiography also showed an abnormal artery (*arrow*)

A 67-year-old man experienced repeated pneumonia during his childhood and often had hemoptysis and bloody phlegm since the age of 38. He always had a cough and sputum during food or liquid intake. During examinations that were conducted before surgery for atrial fibrillation and valvular disease in another hospital, computed tomography (CT) showed severe bronchiectasis in the right middle and lower lung lobes. Due to concerns about the risk of pulmonary bleeding during heart surgery using an artificial cardiopulmonary device with systemic heparinization, the patient was introduced to our hospital for lung resection. The presence of a fistula between the esophagus and the right lower lung lobe was suspected after CT (Fig. 1a), and consequently, esophagoscopy (Fig. 1b) and esophagography (Fig. 1c) through the fistula were performed, which revealed the flow of contrast medium from the esophagus to the right lower lobe and finally to the right main bronchus. CT angiography (Fig. 2a) and catheter angiography (Fig. 2b) revealed an abnormal artery branching from the inferior phrenic artery into the right lower lobe. Endovascular embolization of a few bronchial arteries and the abnormal artery was carried out to reduce bleeding during surgery.

**Fig. 3:**
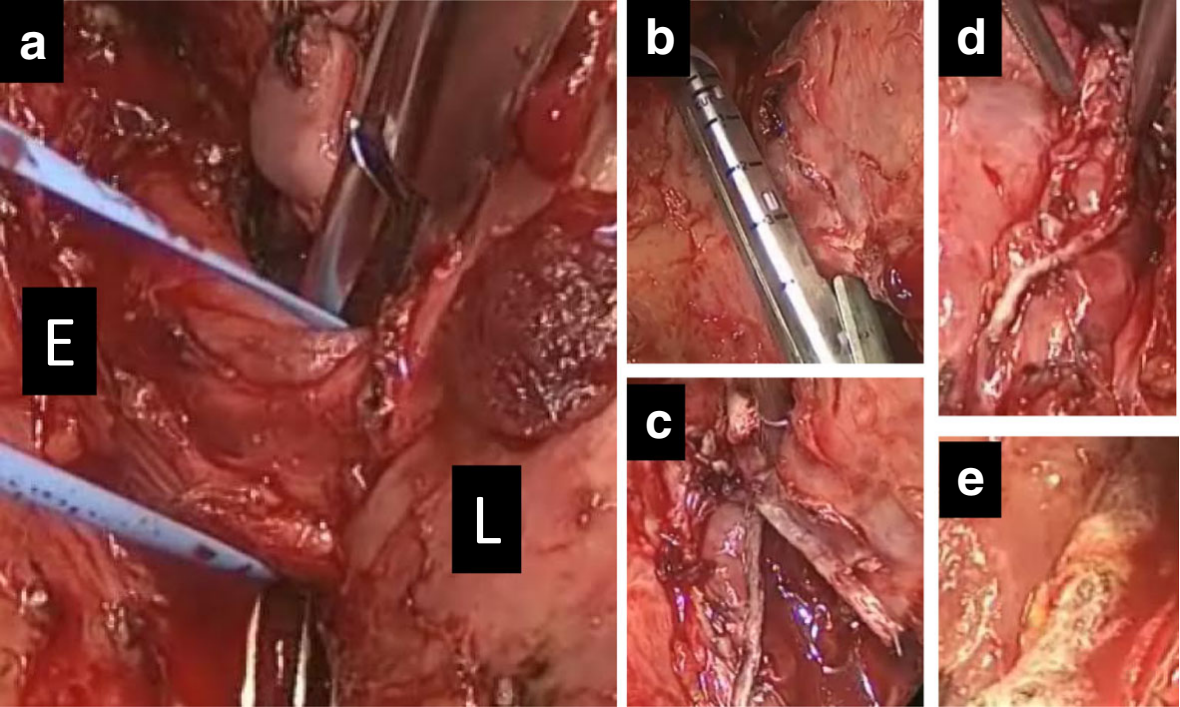
Intraoperative findings (**a**–**e**). **a** Esophagobronchial fistula was pulled up with a tape between the esophagus (E) and the right lower lobe (L). No adhesion was seen and pleura defected around the fistula. **b**, **c** The fistula was divided with an automatic stapler. **d**, **e** Organizations including pleura around the severed esophagus were seamed together and collagen patches coated with human fibrinogen and thrombin (TachoSil) were attached for protection

The patient was placed in the left decubitus position. He underwent thoracotomy through the fifth intercostal space with a posterolateral skin incision. The middle and lower lobes strongly adhered to the chest wall and the diaphragm. We carefully cut the adhesion on the diaphragm using ligation, an energy device, and an automatic stapler because the abnormal artery could have been situated behind it. After that, we located the fistula easily without adhesion to the surroundings, severed it using an automatic stapler, and resected the middle and lower lobes. There was a defect in the pleura around the fistula, and we stitched the surrounding tissue including the pleura and cover it with collagen patches coated with human fibrinogen and thrombin (TachoSil, Nycomed, Vienna, Austria) to strengthen the surface of the esophagus (shown in Fig. 3). Furthermore, we attached the fifth intercostal muscle to the middle and lower bronchus for protection. This surgery took 12 h with bleeding of 4000 ml.

**Fig. 4:**
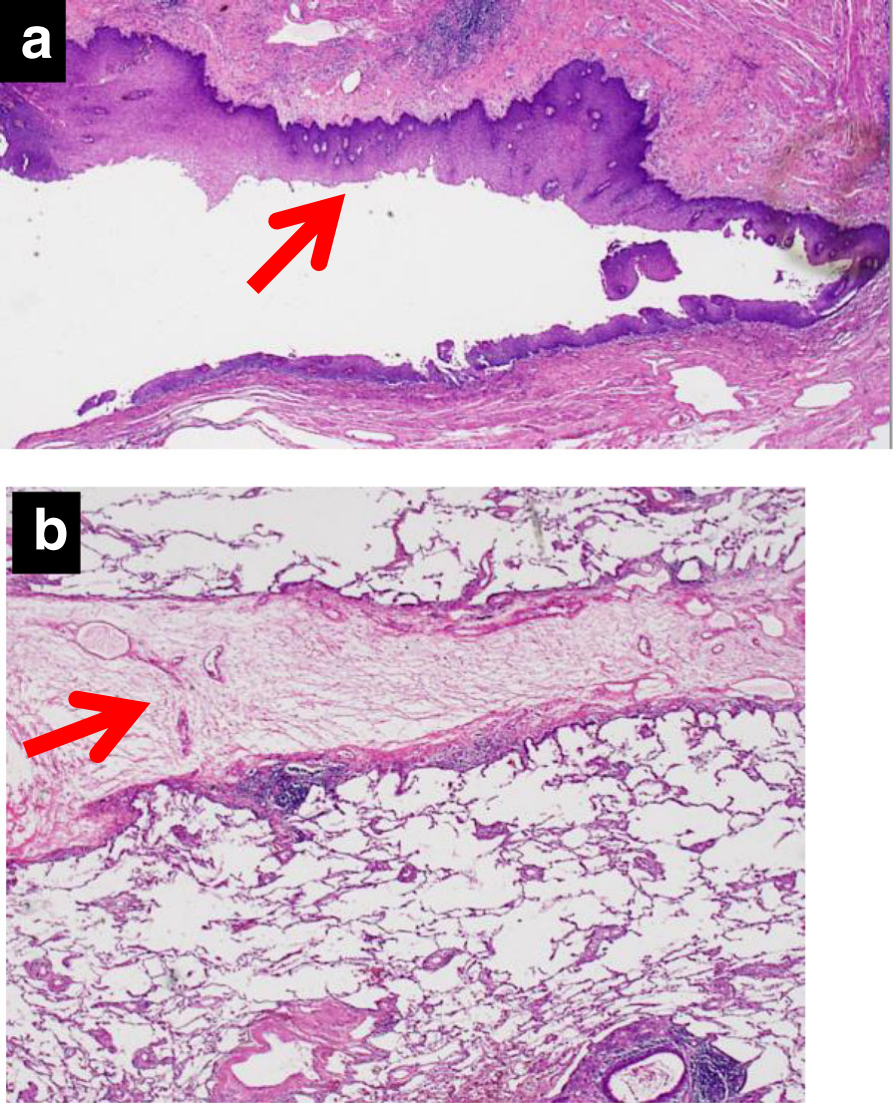
Histopathological findings (×20, hematoxylin and eosin stain). **a** A photomicrographic image shows the fistula from the esophagus to the lung, not directly to the bronchus. The inner wall of the fistula (*arrow*) is covered with esophageal stratified squamous epithelium and bronchial epithelium. There is no evidence of inflammatory change. **b** This microscopic finding may show the septum between the ordinary lung and the sequestrated lung

The diagnosis of congenital EBF to pulmonary sequestration was established with intraoperative and pathological findings (Fig. 4). The symptoms disappeared immediately following the operation, and the patient was discharged on the 12th day after surgery.

### Discussion

Tracheo/bronchoesophageal fistula may be congenital, traumatic, inflammatory, or neoplastic. Most patients with congenital tracheoesophageal fistulas (TEF) are diagnosed immediately following birth or during infancy, because more than 98 % are associated with atresia of the esophagus [1]. Congenital TEF without esophagus atresia H type TEF is rare but reportedly detected in adults because of long-standing respiratory symptoms [1–7]. The distribution of this disease is equal between males and females. It is three times more common on the right than the left and is seen more in the right lower lobe [6, 7]. According to the classification of Braimbridge and Keith [7], type I is a fistula associated with a wide-necked congenital diverticulum of the esophagus with inflammation at the tip. Type II, which is the most common, consists of a short tract running directly from the esophagus to the lobar or segmental bronchi. In type III, the fistulous tract connects the esophagus to a cystic pulmonary change. In type IV, a fistula runs into a sequestered pulmonary segment. The patient described here may come under the definition of type IV because the existence of sequestered lung was suspected on the ground of an abnormal artery from an abdominal artery running into the right lower lung lobe, and a septum was clearly detected in the lobe by microscope.

Symptoms sometimes present during childhood or adulthood but are seldom seen at birth [1, 5–7]. Theories on the cause of this delay have been discussed: a membrane existing on the fistula subsequently ruptures, the fistulous track runs upward and may close during swallowing (the track in this case appeared to run downward), or a fold of esophageal mucosa overlaps the orifice but subsequently becomes less mobile. Symptoms are usually due to chronic bronchopulmonary suppuration, cough (almost universal), hemoptysis, pneumonia, and choking when swallowing liquids (or the appearance of food in the sputum). Although digestive symptoms are relatively uncommon, it was reported that the stomach filled with air on expiration and caused reflux, and dysphagia was sometimes present, 13 and 4 %, respectively [7]. The patient discussed in this paper suffered several episodes of pneumonia during childhood but had not experienced any apparent pulmonary infection in adulthood before the age of 38. Apart from the aforementioned reasons, the silent period could have existed because the tract from the esophagus opened into the sequestered lung, not the bronchus. The reason for the later symptoms that occurred from the age of 38 could be that the continuous inflammation of the sequestered lung led to a connection between the damaged area and the right lower bronchus, and the inflammation was spread over the whole right lower and middle lobes. This theory may support the existence of the sequestered lung, but no image data before the lung got destroyed existed and the theory cannot be established.

The diagnosis is usually made by barium swallow. Bronchoscopy and esophagoscopy sometimes demonstrate the orifices of the fistula, which are usually small and only recognized when the exact site is known [1–7]. In this case, EBF was first suspected by computed tomography, which showed the tract between the esophagus and the right lower lobe, and the heavily damaged right middle and lower lobes.

Criteria for congenital EBF are suggested pathologically by the absence of surrounding inflammation and adherent lymph nodes, along with the presence of mucosa and definitive muscularis mucosa within the fistulous tract. Surgically, uncomplicated and easy dissection of the fistula and absence of inflammation suggests a congenital fistula [7, 8]. In this patient, there was strong adhesion around the destroyed lung, but no adhesion was apparent around the EBF.

In terms of treatment, the division of the fistula and excision of the permanently diseased lung segment have been performed. There was no operative mortality reported, and symptoms rapidly improved [1–7].

## Conclusions

Congenital EBF without esophageal atresia is a differential diagnosis in cases of bronchiectasis in the lower lobe in the elderly.

## Consent

Written informed consent was obtained from the patient for publication of this case report and any accompanying images. A copy of the written consent is available for review by the Editor-in-Chief of this journal.

## Abbreviations

CT: Computed tomography
EBF: Esophagobronchial fistula
